# Supplementary material for: Germline Genetic Findings Which May Impact Therapeutic Decisions in Families with a Presumed Predisposition for Hereditary Breast and Ovarian Cancer
Source: Cancers (Basel). 2020 Aug 3;12(8):2151. doi: 10.3390/cancers12082151 (PMC7465232; doi:10.3390/cancers12082151)
Supplement: Supplementary file 1 [file cancers-12-02151-s001.pdf]

Gene	cDNA change	Protein change	Mutation Type	ExAC	gnomAD	dbSNP	ClinVar	LOVD	ACGM rules	Scaled-CADD-Score
ATM	c.162T>C	p.Y54Y	Silent	0,0016	0,0017	rs3218690	B(5);L.B(5);VUS(1)	B; LB	PM1,BS1,BP6,BP7	
ATM	c.249A>C	p.S83S	Silent	-	-	-	L.B(1)		PM2, BP6,BP7	
ATM	c.348A>G	p.K116K	Silent	-	-	-	-		PM1,PM2,BP7	
ATM	c.1254A>G	p.Q418Q	Silent	0,0007	0,0086	rs4987943	B;L.B	B	BA1,BP6,BP7	
ATM	c.1773T>C	p.N591N	Silent	0,0001	0,0001	rs61734356	B;L.B		PM1,BP6,BP7	
ATM	c.1810C>T	p.P604S	Missense	0,003	0,0031	rs2227922	B(6);L.B(4);VUS(1)	LB	BS1,BS2, BP4	24,3
ATM	c.2289T>A	p.F763L	Missense	0,0005	0,0007	rs34231402	B(1);L.B(3);VUS(1)	LB	PM1,BS1, BP4	16,81
ATM	c.2597T>C	p.V866A	Missense	-	-	-	-		PM1,PM2,BP4	0,001
ATM	c.3078-5T>C	.	Intronic SNV	-	-	-	-		PM2	
ATM	c.3161C>G	p.P1054R	Missense	0,017	0,0163	rs1800057	B	B/LB/VUS	PP3,BS1,BS2,BP6	26,5
ATM	c.3403-15T>A	.	Intronic SNV	0,0026		rs79701258	B/L.B	B/LB	BA1,BP6	
ATM	**c.3663G>A**	**p.W1221***	**Nonsense**	**-**	0	**rs864622490**	**P**	**VUS**	**PVS1,PM2,PP3,PP5**	41
ATM	c.4388T>G	p.F1463C	Missense	0,0013	0,0014	rs138327406	B(6);L.B(4);VUS(3)	B/LB/VUS	PM1,PP3,BS1,BS2	28,7
ATM	c.4396C>G	p.R1466G	Missense	-	-	rs730881369	VUS		PM1,PM2,PP3	29,5
ATM	c.4619A>G	p.D1540G	Missense	-	-	-	-		PM2,BP4	19,03
ATM	c.4654A>G	p.N1552D	Missense	-	-	-	-		PM1,PM2	22,8
ATM	c.4745A>G	p.K1582R	Missense	-	-	-	-		PM1,PM2,PP3	22,1
ATM	c.4776G>A	p.E1592E	Silent	-	-	-	-		PM2	
ATM	**c.4776+2T>C**		**SNVSpliceDonor**	**-**	**-**	**rs587781927**	**P**	**VUS**	**PVS1,PM2,PP3,PP5**	25
ATM	c.5497-15G>C		Intronic SNV	0,0043	0,0043	rs3092828	B	B/LB	BS1,BS2,BP6	
ATM	c.6959A>G	p.D2320G	Missense	-	-	rs1060501640	VUS		PM1,PM2,PP3	23
ATM	c.7187C>G	p.T2396S	Missense	0,0002	0,0001	rs370559102	B(1);L.B(1);VUS(7)	LB	PM1,BS1,BP4	14,35
ATM	c.7831A>G	p.S2611G	Missense	-	-	-	-		PM2,BP4	15,72
ATM	c.7833T>A	p.S2611R	Missense	-	-	-	-		PM2,BP4	13,89
ATM	**c.8934_8935delTG**	**p.E2979Afs*9**	**Frameshift**	**-**	-	**-**	**-**	**P**	**PVS1,PM2**	35
BARD1	c.214A>G	p.S72G	Missense	-	-	-	-		PM1,PM2,PP3,BP1	21,5
BARD1	c.1075_1095del	p.L359_P365del	Deletion In Frame	0,0108	-	rs28997575	B;L.B	B/LB	PM4,BA1,BP6	11,86
BARD1	c.1670G>C	p.C557S	Missense	0,0103	0,015	rs28997576	B;L.B	B	BS1,BS2,BP1,BP4,BP6	0,005
BARD1	c.2212A>G	p.I738V	Missense	0,0077	0,0074	rs61754118	B;L.B	B	BS1,BS2,BP1,BP4,BP6	11,71
BARD1	c.2242G>A	p.Q748K	Missense	-	-	rs879253880	VUS		PM2,BP1,BP4	
BRIP1	**c.484C>T**	**p.R162***	**Nonsense**	**-**	**-**	rs747604569	**P/LP**		**PVS1,PM1,PM2,PP3,PP5**	38
BRIP1	c.508-31C>G	.	Intronic SNV	0,1629	0,1941	rs4988344	B	B	BA1,BP6	
BRIP1	c.550G>T	p.D184Y	Missense	0,0003	-	rs201047375	VUS		PM1,PP3,PP5	25,4
BRIP1	c.577G>A	p.V193I	Missense	0,0057	0,0035	rs4988346	B/L.B	LB	PM1,BS1,BS2,BP4,BP6	0,002
BRIP1	c.728T>C	p.I243T	Missense	-	-	rs587781860	L.B(1);VUS(6)		PM1,PM2,BP4	11,48
BRIP1	c.1341-32C>T	.	Intronic SNV	-	-	-	-		PM2	
BRIP1	c.1935+11_1935+13delGTT	.	DeletionIntronic	-	0,0052	rs730881641	B(2);L.B(2);VUS(2)		BS1	
BRIP1	c.2220G>T	p.Q740H	Missense	0,0005	0,0005	rs45589637	L.B(4);VUS(12)	LB/VUS	PM1,BS1	25,7
BRIP1	c.2637A>G	p.E879E	Silent	0.1849*	0.741	rs49866765	B/L.B	B	BA1,BP6,BP7	
BRIP1	c.2755T>C	p.S919P	Missense	0,3722	0,6006	rs4986764	B/L.B	B/LB	PM1,BA1,BP6,BP7	0,195
BRIP1	c.2905+83T>A		Intronic SNV	0,416	0,3246	rs4988357	-	B	BA1	
BRIP1	c.3411T>C	p.Y1137Y	Silent	0.59412	0,5975	rs4986763	B/L.B	B	BA1,BP6,BP7	
ERCC4	c.33C>T	p.A11A	Silent	0,0134	0,0129	rs3136042	B		PM1,BS1,BS2,BP6,BP7	
ERCC4	c.207+11G>A		Intronic SNV	0,3878	0,2474	rs762521	B		BA1,BP6	
ERCC4	c.207+49G>A		Intronic SNV	0,072	0,616	rs1799798	-		BA1	
ERCC4	c.252C>T	p.L84L	Silent	0,0089	0,0103	rs3136056	B/L.B	B	PM1,BA1,BP6,BP7	
ERCC4	c.388+53dupT		Intronic SNV	-	0,0003	rs34861763	-		BS1	
ERCC4	**c.584+1G>A**		**SNV Splice Donor**	**-**	**-**	**-**	**-**	VUS	**PVS1,PM2,PP3**	25
ERCC4	c.793-69G>T		Intronic SNV	-	0,0103	rs10048099	-		BS1,BS2	
ERCC4	c.974-54delG		DeletionIntronic	-	0,002	rs375946747	-		PM2	
ERCC4	c.974-7G>A		Intronic SNV	-	0,9664	rs254942	B	B	BA1,BP6	
ERCC4	c.1135C>T	p.P379S	Missense	0,0053	0,0046	rs1799802	L.B(1);VUS(2)	LB/VUS	PM1,PP3,BS1,BS2,BP1,BP6	32
ERCC4	c.1244G>A	p.R415Q	Missense	0,0785	0,055	rs1800067	B/L.B		PM1,PP3,BA1,BP1,BP6	35
ERCC4	**c.1251T>A**	**p.C417***	**Nonsense**	**-**	**-**	**rs762738968**	**-**		**PVS1,PM1,PM2,PP3**	36
ERCC4	c.1563C>G	p.S521R	Missense	0,0007	0,0008	rs41552412	VUS		BS1,BP1,BP4	21,8
ERCC4	c.1727G>C	p.R576T	Missense	0,0008	0,0006	rs1800068	VUS	VUS	PP3,BS1,BP1,BP6	24,1
ERCC4	c.1871G>A	p.R624Q	Missense	-	0,0001	rs180919656	-		PM2,PP3,BP1	34
ERCC4	c.1905-28G>A		Intronic SNV	0,2797	0,2387	rs1799800	-		BA1	
ERCC4	c.2117T>C	p.I706T	Missense	0,0022	0,0019	rs1800069	L.B(1);VUS(1)	VUS	PM1,PP3,BS1,BP1,BP6	29,9
ERCC4	c.2505T>C	p.S835S	Silent	0,2943	0,0014	rs1799801	B		BA1,BP6,BP7	
ERCC4	c.2624A>G	p.E875G	Missense	0,0193	0,0131	rs1800124	B/L.B	B	PP3,BS1,BS2,BP1,BP6	27,7
ERCC4	c.2655G>A	p.T885T	Silent	0,0138	0,011	rs16963255	B/L.B	B	BS1,BS2,BP6,BP7	
ERCC4	c.2743A>G	p.K915E	Missense	-	-	-	-		PM2,BP1,BP4	20,5
PALB2	c.-47G>A	.	SNV 5-UTR	0,0236	0,0405	rs8053188	B	B	BA1,BP6	
PALB2	c.49-54C>T	.	Intronic SNV	-	0,0001	rs515726121	L.B	VUS	BS1,BP6	
PALB2	c.212-183T>G	.	Intronic SNV	-	0,0336	rs60253267	L.B		BS1,BS2,BP6	
PALB2	c.212-58A>C	.	Intronic SNV	-	0,0195	rs80291632	B/L.B	B	BS1,BS2,BP6	
PALB2	c.814G>A	p.E272K	Missense	-	0	rs515726127	VUS	LB	PM1,PM2,BP4	12,19
PALB2	c.1010T>C	p.L337S	Missense	0,0192	0,015	rs45494092	B(9);L.B(2);VUS(1)	B/LB/VUS	PM1,BS1,BS2,BP4,BP6	5,275
PALB2	c.1572A>G	p.S524S	Silent	0,0041	0,0031	rs45472400	B/L.B	B/LB/VUS	PM1,BS1,BP6,BP7	
PALB2	c.1676A>G	p.Q559R	Missense	0,0955	0,1072	rs152451	B/L.B	B;LB	PM1,BA1,BP4,BP6	0,001
PALB2	c.1684+39_1684+41delTGA		Intronic deletion	-		rs368593832	L.B	B	BP6	
PALB2	**c.1857delT**	**p.F619Lfs*9**	**Frameshift**	**-**		-	**-**		**PVS1,PM1,PM2,PP5**	35
PALB2	c.2014G>C	p.E672Q	Missense	0,0283	0,0224	rs45532440	B/L.B	B;LB	PM1,BS1,BS2,BP4,BP6	8,057

PALB2	c.2590C>T	p.P864S	Missense	0,0041	0,0027	rs45568339	B/L.B	LB/VUS	PM1,BS1,BP4,BP6	18,44
PALB2	c.2794G>A	p.V932M	Missense	0,0081	0,0051	rs45624036	B/L.B	B/LB/VUS	PM1,BS1,BP4,BP6	26,6
PALB2	c.2816T>G	p.L939W	Missense	0,0015	0,0009	rs45478192	B(5);L.B(8);VUS(4)	LB/VUS	PM1,PP3,BS1,BP6	28
PALB2	c.3114-51T>A		Intronic SNV	0,998	0,9984	rs249936	L.B	B	BS1	
PALB2	c.3300T>G	p.T1100T	Silent	0,0235	0,0235	rs45516100	B/L.B	B/LB/VUS	PM1,BS1,BS2,BP6,BP7	
RAD51C	c.-26C>T		SNV 5-UTR	0,1796	0,1815	rs12946397	L.B	LB	BA1,BP6	
RAD51C	c.572-17G>T		Intronic SNV	0,004	0,004	rs193023469	B;L.B	B/LB/VUS	BS1,BS2,BP6	
RAD51C	c.859A>G	p.T287A	Missense	0,0054	0,0056	rs28363317	B;L.B	B/LB/VUS	PM1,PP3,PP5,BS1,BS2,BP6	26,6
RAD51C	c.838-37G>T		Intronic SNV	-	-	-	-		PM2	
RAD51D	c.83-4T>C		Intronic SNV	-	-	-	L.B.		PM2,BP6	
RAD51D	**c.94_95delGT**	**p.V32Ffs*38**	**Frameshift**	**-**	**0**	**rs786203137**	**P**		**PVS1,PM1,PM2,PP5**	35
RAD51D	c.263+103dupT		Intronic SNV	-	0,106	-	-		BA1	
RAD51D	c.494G>A	p.R165Q	Missense	0,1606	0,1515	rs4796033	B/L.B	B	BA1,BP1,BP4,BP6	16,92
RAD51D	c.629C>T	p.A210V	Missense	0	0,00006	rs376855484	VUS		PP3,BS2,BP1	33
RAD51D	c.695G>A	p.R232Q	Missense	0,0002	0,00448	rs28363283	B/L.B		BS1,BS2,BP1,BP4,BP6	23,2
RAD51D	c.698A>G	p.E233G	Missense	0,0114	0,01090	rs28363284	B/L.B	B	BS1,BS2,BP1,BP4,BP6	24,4

Pathogenic or likely pathogenic variants are indicated in bold
